# Supplementary material for: The Effects of GCSF Primary Prophylaxis on Survival Outcomes and Toxicity in Patients with Advanced Non-Small Cell Lung Cancer on First-Line Chemoimmunotherapy: A Sub-Analysis of the Spinnaker Study
Source: Int J Mol Sci. 2023 Jan 16;24(2):1746. doi: 10.3390/ijms24021746 (PMC9867035; doi:10.3390/ijms24021746)
Supplement: Supplementary file 1 [file ijms-24-01746-s001.zip › ijms-2091024-supplementary.pdf]

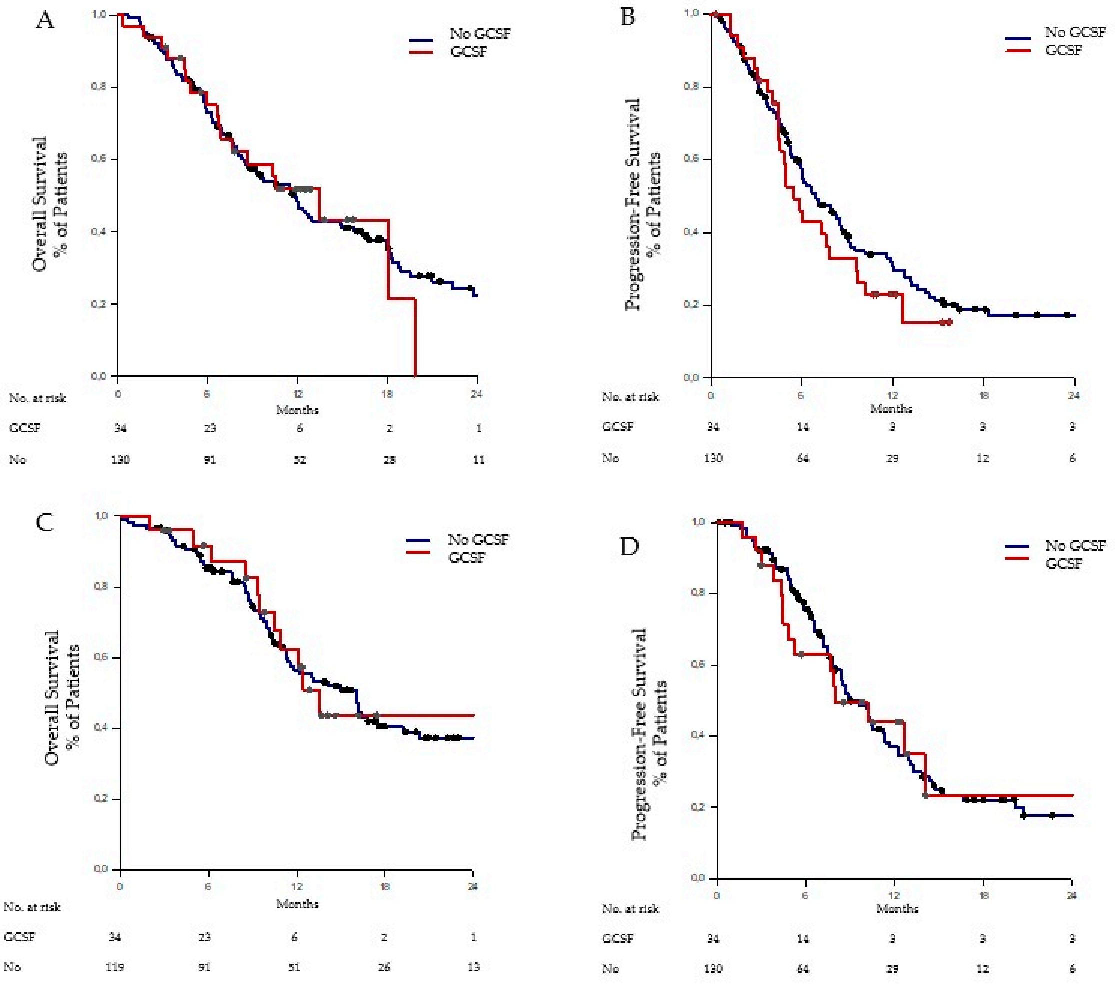

Figure letter (N) NLR	Variable [N - %]	Median OS mo. (95% CI)	p-value (log-rank)	Median PFS mo. (95% CI)	p-value (log-rank)
A/B	GCSF	13.4	0.954	5.5	0.358
(N= 164)	[34 – 21]	(8.0-18.9)		(4.0-7.0)	
NLR High	No GCSF	11.8		6.9	
	[130 – 79]	(9.1-14.5)		(5.0-8.8)	
C/D	GCSF	13.5	0.924	8.0	0.883
(N= 144)	[25 - 17]	(11.1-15.9)		(4.4-11.5)	
NLR low	No GCSF	16.0		9.0	
	[119 - 83]	(12.4-19.7)		(7.3-10.7)	

Abbreviations: aNSCLC, advanced non-small-cell-lung cancer; CI, confidence interval; NLR, neutrophil-to-lymphocyte ratio (cut-off ≥ 4); OS, overall survival; PFS, progression-free survival

Supplementary Figure S1. OS and PFS by GCSF use in patients with aNSCLC treated with chemoimmunotherapy according to NLR.
